# Evaluation of Interfacial Adhesion Properties Between Nano-Modified Composite Polyurethane and Cement-Stabilized Macadam Based on Surface Free Energy

**DOI:** 10.3390/ma19122458

**Published:** 2026-06-08

**Authors:** Hongwei Fu, Zhifeng Chu, Sha Wang, Haiwei Gao, Nanxiang Zheng, Zeyu Liu

**Affiliations:** 1 Shaanxi Transportation Holding Group Co., Ltd., Xi’an 710064, China; 15991753680@163.com (H.F.); chzhf1975@126.com (Z.C.); 13992877529@163.com (S.W.); 2 Sichuan Chuanwen Expressway Co., Ltd., Chengdu 610041, China; hillware@sina.com; 3 School of Highway, Chang’an University, Xi’an 710064, China; emailznx@163.com

**Keywords:** composite polyurethane, cement-stabilized macadam, bonding performance, surface free energy, image recognition

## Abstract

The non-excavation grouting technology of composite polyurethane materials provides an efficient and economical treatment scheme for deep-seated distresses of asphalt pavements. To quantitatively evaluate the bonding performance between composite polyurethane and cracks in cement-stabilized macadam base, *Image J(v1.54p) (National Institutes of Health, Bethesda, MD, USA)* image recognition was adopted to analyze the gradation at the interface of cement-stabilized macadam base mixture. The surface free energy theory was applied to quantitatively study the work of adhesion at the interface between three types of nano-modified composite polyurethane materials (G1-2, T-1 (0.1 wt% MWCNTs and 0 NanoSiO_2_@KH550), and TG-1 (0.5 wt% MWCNTs and 0.5 wt% NanoSiO_2_@KH550)) and cement-stabilized macadam mixture, and predict the interfacial bonding performance. The results showed the bonding performance order as T-1 > G1-2 > TG-1. In addition, the micro-interface between nano-modified composite polyurethane materials and cement-stabilized macadam base was analyzed via SEM images, revealing the bonding mechanism at the interface between them.

## 1. Introduction

During the long-term service of asphalt pavements, cement-stabilized macadam bases are subject to multiple impact loads such as repeated vehicle loads, environmental erosion and material aging, leading to frequent occurrence of deep crack distresses [1]. If such cracks are not treated effectively and in a timely manner, they will expand rapidly and induce chain distresses such as pavement settlement, pumping and alligator cracking, which greatly shortens the service life of pavements and increases maintenance costs [2]. Although traditional excavation repair technologies can alleviate distresses temporarily, they have disadvantages such as long construction period, great traffic interference and high resource consumption, which are difficult to meet the high-efficiency maintenance requirements of modern highways [3].

Composite polyurethane materials have shown significant advantages in the non-excavation grouting repair of deep cracks in asphalt pavements due to their excellent fluidity, rapid curing property and high bonding strength [4,5,6,7]. The materials can penetrate into the cracks of cement-stabilized macadam base through grouting equipment, fully fill the pores and form an integral stress structure with the base, effectively restoring pavement bearing capacity and improving waterproof performance [8]. However, in the actual service environment, the interfacial adhesion failure between grouting materials and cement-stabilized macadam base occurs frequently—moisture intrusion, temperature cycles and fatigue loads will gradually weaken the interfacial bonding strength, leading to re-cracking in the repaired area and seriously affecting the treatment effect [9]. Interfacial adhesion performance has become the core factor determining the durability of grouting repair, but there are still obvious deficiencies in its quantitative evaluation and mechanism research [10,11].

Traditional evaluation methods for interfacial adhesion performance have significant limitations. Macro mechanical tests, including both general strength tests (compression, tension, flexural tensile tests) and direct bonding tests (peel, pull-off, slant shear tests), only reflect the overall mechanical properties of the composite system. They fail to reveal the micro interfacial mechanism and quantitatively explain the essential causes of adhesion failure [12]; common adhesion evaluation methods such as the boiling water test and the water immersion test are simple to operate but rely on subjective judgment and lack quantitative indicators, especially with insufficient evaluation accuracy for the interfacial performance of nano-modified grouting materials [13,14]. Although Yu Guohong et al. realized semi-quantitative evaluation by extending boiling time and introducing mass loss rate [15], and Fan Liang et al. optimized the evaluation method by calculating spalling area with image analysis software [16], these improvements still do not deviate from the empirical test framework and fail to establish the correlation between adhesion performance and material characteristics from the perspective of thermodynamic essence.

The development of surface free energy theory provides a new approach for the quantitative evaluation of interfacial adhesion performance. The theory holds that the bonding strength of material interfaces essentially depends on the intermolecular interactions on the surface, which can be accurately characterized by thermodynamic indicators such as surface free energy parameters (dispersion component, polar component), work of adhesion and work of debonding [17]. In 2002, Cheng et al. first evaluated the adhesion by measuring the surface free energy of asphalt and aggregates, using the absolute ratio of the Gibbs free energy change in the system under anhydrous and aqueous conditions, and the results were highly consistent with the conclusions of accelerated water damage tests [18]; Xiao Qingyi et al. combined wetting-adsorption theory with SEM observation and confirmed that the work of adhesion could effectively replace the boiling water test to characterize the bonding characteristics of the asphalt–aggregate interface [19]; Liu Yamin et al. measured the surface energy parameters of SBS modified asphalt and different aggregates, and clarified that the SBS-amphibole gneiss system had the optimal adhesion [20]; Chen Yanjuan et al. found that adding anti-stripping agent could improve the polar component of asphalt surface energy, and the work of debonding had a good correlation with the TSR value of freeze–thaw splitting test [21]. In recent years, Hamedi et al. verified the correlation between surface energy parameters and the tensile strength of asphalt–aggregate interface through tensile tests [22], and scholars such as Wang Lan and Han Sen further applied this theory to the study of interfacial performance of aged asphalt and lime-modified asphalt, providing theoretical support for the optimal design of modified asphalt materials [23,24].

Although the surface free energy theory has achieved rich results in the research of asphalt–aggregate interface, there are still obvious gaps in the research on the interface between nano-modified composite polyurethane and cement-stabilized macadam base: on the one hand, most existing studies focus on asphalt materials, with insufficient discussion on the surface free energy characteristics and interfacial mechanism of organic–inorganic composite grouting materials such as composite polyurethane; on the other hand, the introduction of nano-modifiers (such as carbon nanotubes and nano-SiO_2_) will change the surface molecular structure and micro-morphology of grouting materials, and the its influence law on interfacial work of adhesion and water resistance to spalling has not been clarified. In addition, the lack of a weighted calculation model based on the composition ratio of the three phases (aggregate, cement mortar, pore) of cement-stabilized macadam makes it difficult to truly reflect the influence of the heterogeneous structure of the base on interfacial performance [25]. Furthermore, most existing studies are based on qualitative analysis, failing to systematically establish the quantitative relationship between surface energy evaluation indicators and traditional adhesion indicators, which cannot provide an accurate basis for the formula optimization of nano-modified grouting materials.

Based on this, this paper takes the interfacial adhesion performance between nano-modified composite polyurethane grouting materials and cement-stabilized macadam base as the research core, aiming to solve the following key problems: (1) establish a quantitative analysis method for the three-phase composition ratio of cement-stabilized macadam based on *Image J(v1.54p)* image recognition and t-distribution statistics, providing basic data for interfacial thermodynamic calculation [26]; (2) determine the surface free energy parameters of three nano-modified composite polyurethanes (G1-2, T-1, TG-1) and cement-stabilized macadam components (limestone aggregate, cement mortar) through contact angle tests, calculate the interfacial work of adhesion and work of debonding and energy ratio, and realize the quantitative evaluation of adhesion performance and water stability [27]; (3) reveal the interfacial bonding mechanism combined with SEM micro-observation, and clarify the synergistic mechanism of chemical bonds, molecular forces and mechanical forces [28,29]. The research results can provide a theoretical basis and technical support for the formula optimization of nano-modified grouting materials and the efficient repair of cracks in cement-stabilized macadam base.

## 2. Test Materials and Research Methods

### 2.1. Test Materials

The nano-modified composite polyurethane grouting materials selected in this chapter are: KH550-grafted nano-SiO_2_ modified composite polyurethane material (specimen No.: G1-2), MWCNTs filler modified composite polyurethane material (specimen No.: T-1) and KH550-grafted nano-SiO_2_ compounded with carbon nanotube modified composite polyurethane material (specimen No.: TG-1). KH550 was obtained from Xi’an Qiyue Biotechnology Co., Ltd. (Xi’an, China), nano-SiO_2_ from Xi’an Funcmater New Material Technology Co., Ltd. (Xi’an, China), MWCNTs from Xi’an Bona Material Technology Co., Ltd. (Xi’an, China), and polyurethane raw materials from Shaanxi Chenxiyi Industrial Co., Ltd. (Xi’an, China). The detailed composition, preparation conditions, and relevant performance parameters of the aforementioned materials summarized in Table 1 [30,31,32].

**Table 1:** List of test raw materials.

Raw Material Name (Abbreviation)	Specification	Technical Index
WGPU@SiO_2_ (G1-2)	0.3 wt% (NanoSiO_2_@KH550)	Table 2
WGPU@CNT (T-1)	0.5 wt% (MWCNTs)	Table 2
WGPU@CNT&SiO_2_ (TG-1)	0.5 wt% (MWCNTs) + 0.1 wt% (NanoSiO_2_@KH550)	Table 2
Cement-stabilized macadam base specimen	φ150 mm × 150 mm	Table 3, Table 4 and Table 5

**Table 2:** Technical indexes of nano-modified composite polyurethane materials.

Performance Index	Unit	G1-2	T-1	TG-1	Required Value
Gel time	s	115	120	110	≥20
Curing time	s	520	530	510	≤800
Viscosity (25 °C)	mPa·s	318	329	333	≤1000
Compressive strength (25 °C)	MPa	31.87	29.14	29.65	≥15
Tensile strength (25 °C)	MPa	8.78	7.25	8.17	≥2
Bonding strength (25 °C)	MPa	2.31	2.38	2.25	≥2
Expansion ratio	—	1.14	1.12	1.12	1~20
Environmental protection	—	Qualified	Qualified	Qualified	No water pollution
Heavy metal	—	Qualified	Qualified	Qualified	Meet standard sanitary requirements

The aggregate of raw materials for cement-stabilized macadam base specimens (CSMs) is limestone. The aggregate specifications are determined as G2, G8, G11 and XG3 with reference to the Technical Guidelines for Construction of Highway Road Bases [1], as shown in Table 3 in detail.

The sieving results of each specification of the four grades of aggregate by the water washing method are shown in Table 4.

Ordinary 42.5 Portland cement was used with a cement dosage of 6% by dry mass, the mixture water content was controlled at 5.0%, and all measured values met the specification requirements. The technical parameters are shown in Table 5.

### 2.2. Preparation of Cement-Stabilized Macadam Base Specimens

In accordance with Test Methods of Materials Stabilized with Inorganic Binders for Highway Engineering (JTG E51-2009) [2], the heavy compaction test was carried out. Cylindrical cement-stabilized macadam base specimens with a size of φ150 mm × 150 mm were prepared according to the C-B-3 compaction scheme for the gradation mixture of cement-stabilized macadam base of expressways. The specimens were cured in a constant temperature and humidity standard curing box with a temperature of 20 ± 2 °C and a humidity of more than 95%.

### 2.3. Test Methods

The interface performance between G1-2, T-1, TG-1 modified composite polyurethane composites and cement-stabilized macadam base can be evaluated by the weighted calculation of the composition ratio (aggregate, cement mortar and pores) of cement-stabilized macadam mixture. The tests mainly include the analysis of cement-stabilized macadam mixture composition ratio, contact angle test and SEM characterization.

(1) Analysis of cement-stabilized macadam mixture composition ratio

The φ150 mm × 150 mm cylindrical cement-stabilized macadam base specimens were cut, and the image information of the cross-sections of the cut specimens was collected. The cross-section images were processed and analyzed by *Image J(v1.54p)* software, and the proportions of coarse aggregate, cement mortar and pores in the cement-stabilized macadam mixture were obtained through statistical calculation.

1. Collection of original cross-section images of cement-stabilized macadam mixture

**Figure 1:**
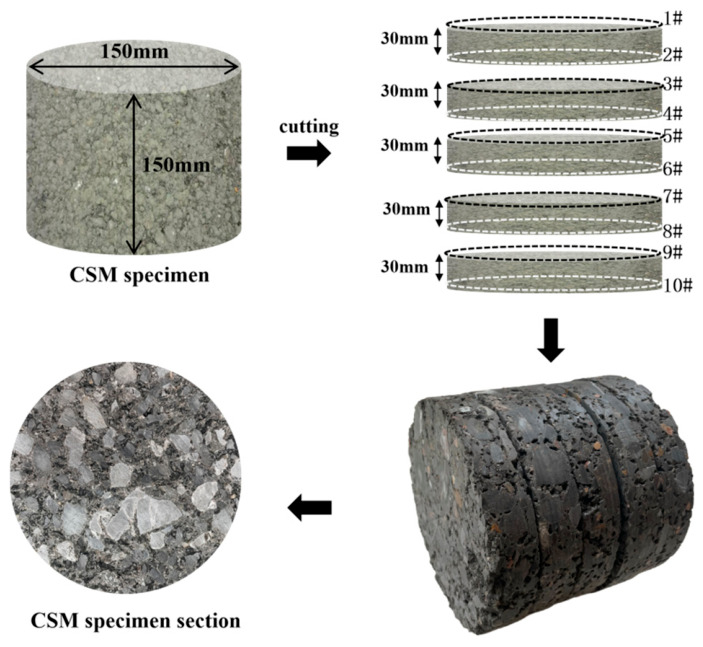
Schematic diagram of collecting original cross-section images of cement-stabilized macadam mixture specimens.

As shown in Figure 1, the φ150 mm × 150 mm cylindrical cement-stabilized macadam base specimen was cut into 5 cylinders with equal thickness, the top and bottom surface images of the cut surface of each cylindrical specimen were collected by high-definition image equipment, and the cross-section images of No.1~No.10, as shown in Figure 1, were obtained.

In general, the image information of the two cross-sections of the same cut surface should be the same. However, in the process of cutting the specimen, due to the inhomogeneity of the aggregate in the cement-stabilized macadam mixture, the combined action of the blade and water may lead to slight differences in the image information of the two different cross-sections of the same cut surface. Therefore, when calculating the composition ratio, the images of both cross-sections need to be collected and statistically calculated.

2. Image processing and analysis of cylindrical cut surfaces

**Figure 2:**
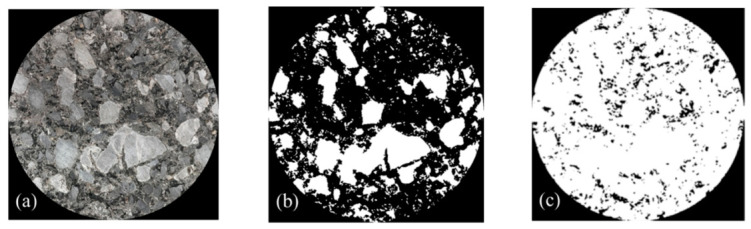
Schematic diagram of original cross-section image processing of cement-stabilized macadam mixture ((**a**) original image, (**b**) limestone aggregate (white), (**c**) pores (black)).

The cross-section images were processed and analyzed by *Image J(1.54p)* software through conversion, sharpening, smoothing, segmentation and other methods. The area information of aggregate, cement mortar and pores was extracted from the binary images after threshold filtering, as shown in Figure 2. Specifically, images were converted to 8-bit grayscale, then smoothed using a Gaussian blur filter (σ = 1.0) and sharpened (radius = 1.0, amount = 0.6) to improve contrast. Segmentation was performed via thresholding, with regions of interest selected based on distinct grayscale differences. A fixed 500 μm × 500 μm window centered on the cross-section was used to calculate the area fractions of each component, excluding edge artifacts.

The composition ratios of coarse aggregate, cement mortar and pores in the cement-stabilized macadam base mixture (i.e., the area fractions of each phase) were calculated according to Equations (1)–(3), which were used as the basic data for calculating the surface free energy of the interface between polyurethane composites and cement-stabilized macadam mixture.

(1) $$p_{i}=\bar{p_{i}}-\frac{t_{\alpha }}{\sqrt{n}}\sigma_{i}$$

(2) $$\bar{p_{i}}=\sum_{j=1}^{n}p_{ij}$$

(3) $$\sigma_{i}=\sqrt{\frac{1}{n}\sum_{j=1}^{n}{\left(p_{ij}-\bar{p_{i}}\right)}^{2}}$$

where $p_{i}$ is the representative area value (i = 1, 2, 3; 1 for limestone aggregate, 2 for cement mortar, 3 for pores);

$\bar{p_{i}}$ is the average area value (i = 1, 2, 3);

$\sigma_{i}$ is the area standard deviation (i = 1, 2, 3);

$p_{ij}$ is the area (i = 1, 2, 3; j = 1~10, representing the serial number of cross-section images);

n = 10, the maximum serial number of collected image cross-sections;

$t_{\alpha }$ is the t-value with a guaranteed rate of 95%.

(2) Contact angle test

The sessile drop method was adopted to measure the contact angles of composite polyurethane composites (G1-2, T-1, TG-1), limestone aggregate, and cement mortar with two standard liquids: distilled water and diiodomethane, with a drop volume of 3 μL. To accurately solve the OWRK model and separately obtain the polar and dispersive components of solid surface energy, two probe liquids with completely different polar characteristics are required. Distilled water with strong polarity and diiodomethane with nearly non-polar properties form the most classic and standardized liquid combination for OWRK surface energy calculation, which has been widely adopted in material surface wettability research, were used [33,34]. The surface free energy, polar component, and dispersive component of each liquid are listed in Table 6.

Before the test, all samples were polished with 2000-grit sandpaper to ensure a flat surface, then ultrasonically cleaned with ethanol for 10 min and rinsed with deionized water to remove surface contaminants. The samples were then dried in an oven at 40 °C for 24 h and stored in a desiccator to avoid moisture absorption before the contact angle measurement. For the limestone aggregate and cement mortar specimens, the preparation process included cleaning, cutting into ~1 cm thick flakes, sequential polishing with #400-, #800-, and #1200-grit sandpaper to obtain a smooth, uniform surface, rinsing with ethanol, and oven-drying at 105 °C to a constant mass to eliminate moisture effects.

To minimize the absorption of probe liquids by the porous structure, the contact time was kept within 5 s, and the measurement was completed before significant penetration occurred. For each material, at least five measurements were taken at different positions on the surface, and the average contact angle was calculated to reduce the influence of local surface roughness and ensure data reliability.

The measured contact angles were then used to calculate the surface free energy of each material and its polar and dispersive components, based on the Owens–Wendt–Rabel–Kaelble (OWRK) model [33]. The core equation of the OWRK model is shown in Equation (4):

(4) $$\gamma_{l}\left(1+\cos\theta \right)=2\left(\sqrt{\gamma_{s}^{d}\gamma_{l}^{d}}+\sqrt{\gamma_{s}^{p}\gamma_{l}^{p}}\right)$$

where $\gamma_{l}$ is the surface free energy of the test liquid (mJ/m^2^);

θ is the measured contact angle (°);

$\gamma_{l}^{d}$ is the dispersive component of the liquid (mJ/m^2^);

$\gamma_{l}^{p}$ is the polar component of the liquid (mJ/m^2^);

$\gamma_{s}^{d}$ is the dispersive component of the solid surface free energy (to be solved, mJ/m^2^);

$\gamma_{s}^{p}$ is the polar component of the solid surface free energy (to be solved, mJ/m^2^);

This equation allows the calculation of both the dispersive and polar components of the solid surface energy using the known liquid parameters and the measured contact angles. These surface energy components are then used as inputs in the subsequent adhesion work calculations.

(3) Adhesion work between composite polyurethane and cement-stabilized macadam mixture

Cement-stabilized macadam mixture is composed of limestone aggregate, cement mortar and pores. When G1-2, T-1 and TG-1 are used to treat the cracks of cement-stabilized macadam base, they bond with the three components. The interaction between composite polyurethane composites and pores is regarded as the cohesion of composite polyurethane composites. Therefore, using the surface free energy components obtained from the contact angle test via the OWRK model as inputs, the adhesion work of G1-2, T-1 and TG-1 with limestone aggregate, cement mortar and pores was calculated, respectively, according to Equations (5)–(7). The overall adhesion work of each polyurethane composite with the cement-stabilized macadam mixture was then calculated by weighting the individual adhesion work values by the composition ratio of each phase in the mixture.

(5) $$W_{wp}=2\gamma_{wp}=2(\gamma_{wp}^{d}+\gamma_{wp}^{p})$$

(6) $$W_{wpl}=2(\sqrt{\gamma_{wp}^{d}\gamma_{l}^{d}}+\sqrt{\gamma_{wp}^{p}\gamma_{l}^{p}})$$

(7) $$W_{wpm}=2(\sqrt{\gamma_{wp}^{d}\gamma_{m}^{d}}+\sqrt{\gamma_{wp}^{p}\gamma_{m}^{p}})$$

where $W_{wp}$ is the cohesion work of composite polyurethane composites (mJ/m^2^);

$W_{wpl}$ is the adhesion work between composite polyurethane and limestone (mJ/m^2^);

$W_{wpm}$ is the adhesion work between composite polyurethane and cement mortar (mJ/m^2^);

$\gamma_{wp}$ is the surface free energy of composite polyurethane composites (mJ/m^2^);

$\gamma_{wp}^{d}$ is the dispersion component of surface free energy of composite polyurethane composites (mJ/m^2^);

$\gamma_{wp}^{p}$ is the polar component of surface free energy of composite polyurethane composites (mJ/m^2^);

$\gamma_{l}^{d}$ is the dispersion component of surface free energy of limestone (mJ/m^2^);

$\gamma_{m}^{d}$ is the dispersion component of surface free energy of cement mortar (mJ/m^2^);

$\gamma_{l}^{p}$ is the polar component of surface free energy of limestone (mJ/m^2^);

$\gamma_{m}^{p}$ is the polar component of surface free energy of cement mortar (mJ/m^2^).

(4) Spalling work

Water is an important factor causing cracking and accelerated deterioration of pavement structure. Spalling work can quantitatively describe the energy required for composite polyurethane to fall off from the surface of cement-stabilized macadam mixture in the cracks of asphalt pavement structure under the action of water. Using the surface free energy components and adhesion work values obtained from the previous contact angle test as inputs, the spalling work of G1-2, T-1 and TG-1 with limestone aggregate, cement mortar and pores was calculated according to Equations (8) and (9), and then the spalling work of G1-2, T-1 and TG-1 with cement-stabilized macadam mixture was calculated based on the weight of the composition ratio of cement-stabilized macadam mixture.

(8) $$\begin{array}{cc} W_{wpwl} & =W_{wpl}-W_{wpw}-W_{lw}\\ & =-2\gamma_{w}+2(\sqrt{\gamma_{wp}^{d}\gamma_{l}^{d}}+\sqrt{\gamma_{wp}^{p}\gamma_{l}^{p}}-\sqrt{\gamma_{wp}^{d}\gamma_{w}^{d}}-\sqrt{\gamma_{wp}^{p}\gamma_{w}^{p}}\\ & -\sqrt{\gamma_{l}^{d}\gamma_{w}^{d}}-\sqrt{\gamma_{l}^{p}\gamma_{w}^{p}}) \end{array}$$

(9) $$\begin{array}{cc} W_{wpwm} & =W_{wpm}-W_{wpw}-W_{mw}\\ & =-2\gamma_{w}+2(\sqrt{\gamma_{wp}^{d}\gamma_{m}^{d}}+\sqrt{\gamma_{wp}^{p}\gamma_{m}^{p}}-\sqrt{\gamma_{wp}^{d}\gamma_{w}^{d}}-\sqrt{\gamma_{wp}^{p}\gamma_{w}^{p}}\\ & -\sqrt{\gamma_{m}^{d}\gamma_{w}^{d}}-\sqrt{\gamma_{m}^{p}\gamma_{w}^{p}}) \end{array}$$

where $W_{wpwl}$ is the spalling work between composite polyurethane and limestone (mJ/m^2^);

$W_{wpwm}$ is the spalling work between composite polyurethane and cement mortar (mJ/m^2^);

$W_{wpw}$ is the adhesion work between composite polyurethane and water (mJ/m^2^);

$W_{lw}$ is the adhesion work between limestone and water (mJ/m^2^);

$W_{mw}$ is the adhesion work between cement mortar and water (mJ/m^2^);

$\gamma_{w}^{d}$ is the dispersion component of surface free energy of water (mJ/m^2^);

$\gamma_{w}^{p}$ is the polar component of surface free energy of water (mJ/m^2^).

(5) Energy ratio of composite polyurethane–cement-stabilized macadam mixture (limestone and cement mortar)

Energy ratio is a reliable method to evaluate the water stability of the bonding interface between composite polyurethane and cement-stabilized macadam mixture. A larger energy ratio value indicates better resistance to water damage of the bonding interface. The energy ratio index is the ratio of bonding work under dry conditions to spalling work under water environment. The energy ratios of composite polyurethane (G1-2, T-1, TG-1)–cement-stabilized macadam mixture (limestone and cement mortar) were calculated according to Equation (10), using the adhesion work and spalling work values obtained from previous calculations as inputs.

(10) $$ER=\frac{aW_{wpl}+bW_{wpm}+cW_{wp}}{aW_{wpwl}+bW_{wpwm}}$$

where ER is the energy ratio of composite polyurethane–cement-stabilized macadam mixture;

a is the proportion of limestone aggregate in cement-stabilized macadam mixture (%);

b is the proportion of cement mortar in cement-stabilized macadam mixture (%);

c is the porosity of cement-stabilized macadam mixture (%);

the other symbols are the same as above.

(6) Scanning electron microscopy (SEM) characterization

The micro-morphological characteristics of the interface between composite polyurethane and cement-stabilized macadam matrix were analyzed by scanning electron microscopy (SEM) (KYKY Technology, Beijing, China). Before observation, the samples were dried in an oven at 60 °C for 24 h to remove residual moisture. Subsequently, they were fractured to expose the bonding interface, then sputter-coated with a thin layer of gold (approximately 10 nm) under vacuum conditions to improve electrical conductivity. SEM imaging was performed using a field-emission scanning electron microscope at an accelerating voltage of 15 kV, with magnifications ranging from 100× to 5000× to observe both the overall interface morphology and local microstructural features.

## 3. Test Results and Discussion

### 3.1. Proportions of Aggregate, Cement Mortar and Pores in Cement-Stabilized Macadam Mixture

**Figure 3:**
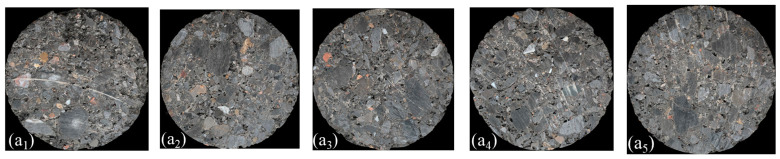

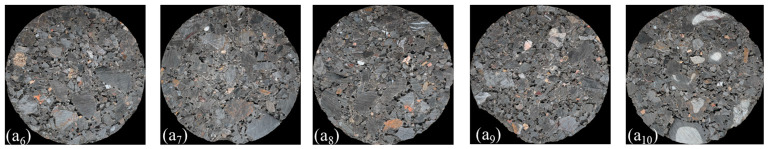
Original images of cut surfaces of cement-stabilized macadam base specimens (cross-section serial numbers (**a1**–**a10**)).

**Figure 4:**
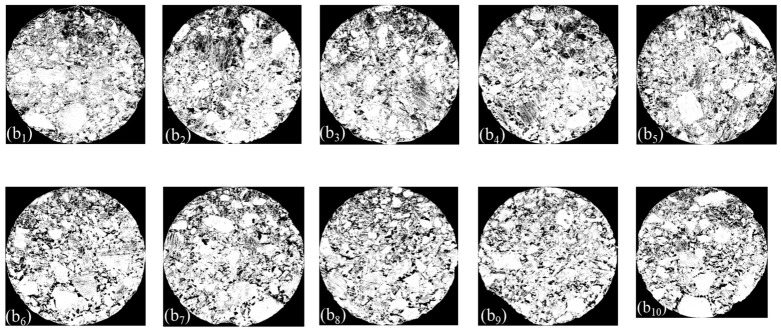
Extracted limestone aggregate regions (white) from cement-stabilized macadam base specimens (cross-section serial numbers (**b1**–**b10**)).

**Figure 5:**
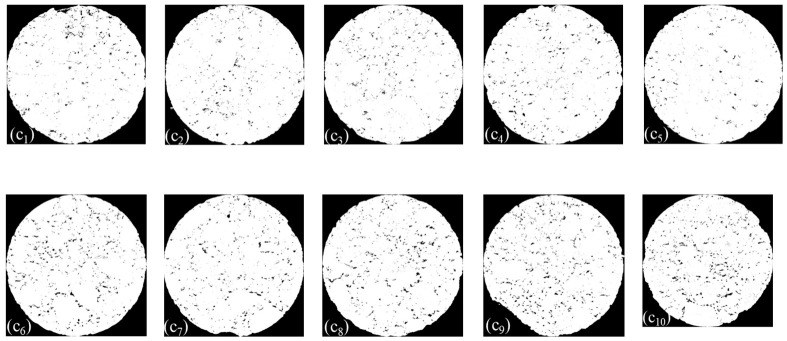
Extracted pore regions (black) from cement-stabilized macadam base specimens (cross-section serial numbers (**c1**–**c10**)).

The image information of 10 cut surfaces was collected, and processed and analyzed by *Image J(v1.54p)* software. Binary images were obtained after smoothing, threshold filtering and other processes, and the area information of limestone aggregate, cement mortar and pores was extracted, as shown in Figure 3, Figure 4 and Figure 5.

**Figure 6:**
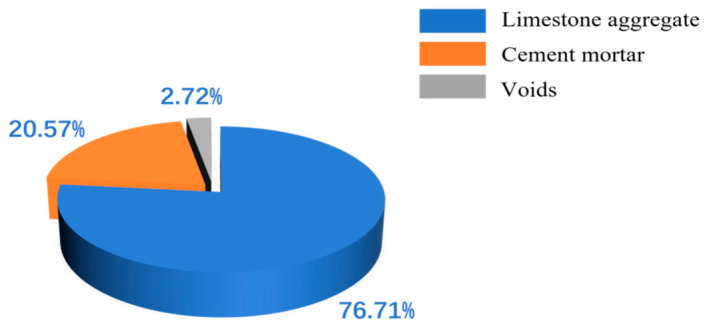
Composition ratio of cement-stabilized macadam mixture (limestone aggregate: 76.71%, cement mortar: 20.57%, pores: 2.72%).

According to the area information of limestone aggregate, cement mortar and pores extracted by *Image J(v1.54p)* software, the representative area values of the three phases of cement-stabilized macadam base mixture were calculated according to Equations (1)–(3) with the parameters set as 95% confidence interval and t-distribution with a degree of freedom of 9, and the composition ratios of the three phases (limestone aggregate, cement mortar and pores) in cement-stabilized macadam mixture were obtained by conversion, as shown in Figure 6.

### 3.2. Evaluation of Interface Adhesion Performance Between Modified Composite Polyurethane and Cement-Stabilized Macadam

(1) Surface free energy

1. Surface free energy of three nano-modified composite polyurethane composites

**Figure 7:**
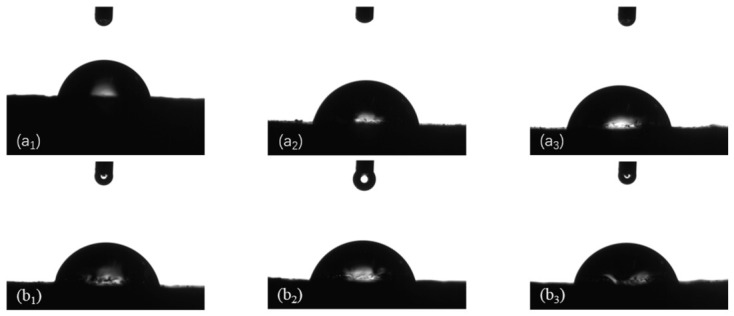

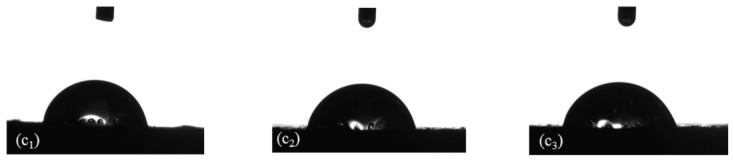
Contact angles of composite polyurethane composites with distilled water (ai: G1-2; bi: T-1; ci: TG-1).

**Figure 8:**
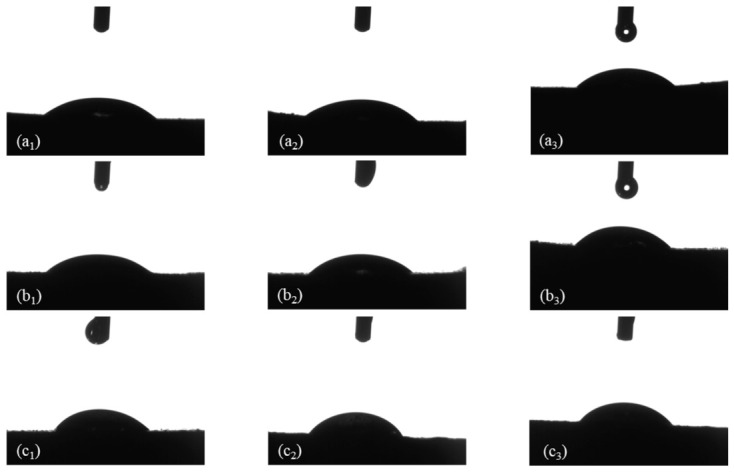
Contact angles of composite polyurethane composites with diiodomethane (ai: G1-2; bi: T-1; ci: TG-1).

Figure 7 and Figure 8 are the contact angle test images of three nano-filler modified composite polyurethane composites (G1-2, T-1, TG-1) with distilled water and diiodomethane, respectively. It can be seen from the figures that, among the contact angles between the two known standard liquids (distilled water and diiodomethane) and different composite polyurethane composites, the contact angle between distilled water and TG-1 composite polyurethane composite is the largest, and the contact angles of the three nano-modified composite polyurethane composites are all close to the right angle, indicating that the three nano-modified composite polyurethane composites all have certain hydrophobicity.

The average values and variation coefficients of the contact angles (n = 5 replicates per material) of the three nano-modified composite polyurethane composites with the two standard liquids were calculated, and the results are listed in Table 7. It can be seen from the calculation results that the variability of the measured contact angles of each group of nano-modified composite polyurethane composite samples is less than 5%, indicating that the data measured in the test are relatively accurate and have good reliability.

In the contact angle test, when different standard liquids are used to test solids, there is a linear relationship between the surface free energy of the standard liquid and the contact angle. The surface free energy of the three nano-modified composite polyurethane composites was calculated, and the calculation results are shown in Table 8. All contact angle data were obtained from five repeated tests, and the data fluctuation is small.

As shown in Table 8, the surface free energy of the three nano-modified composite polyurethanes is in the order of G1-2 > T-1 > TG-1. Error propagation analysis was performed based on the contact angle data, and a t-test confirmed significant differences among groups at the 95% confidence level. It can be seen from the test results that the dispersion component in the surface free energy is significantly higher than the polar component, indicating that the intermolecular interaction on the surface of the three nano-modified composite polyurethane composites is strong, their compatibility with non-polar substances is better, the contact angle is reduced, and the wettability is enhanced, which is conducive to improving the bonding performance of the bonding interface.

2. Surface free energy of limestone aggregate and cement mortar

The contact angle test results of limestone aggregate and cement mortar with distilled water and diiodomethane are shown in Table 9.

It can be seen from Table 9 that the variability of the contact angle test data is about 5%, which can be used as the basis for calculating the surface free energy. All contact angle measurements were performed with five replicates per material, ensuring the reliability of subsequent calculations. The surface free energy of limestone aggregate and cement mortar was calculated, and the calculation results are shown in Table 10.

It can be seen from Table 10 that the dispersion component of limestone aggregate is significantly higher than its polar component, indicating that limestone aggregate has good compatibility with the three nano-modified composite polyurethane composites (G1-2, T-1, TG-1), and the three composites have good wettability with limestone, which is conducive to improving the bonding performance of their bonding interface. These values are consistent with typical ranges reported in the literature for limestone aggregates and cement mortar under similar conditions. The low coefficients of variation (<5%) confirm the reliability of the measured data [35].

The dispersion component and polar component of cement mortar are similar, and its surface free energy is larger than that of limestone aggregate. Therefore, increasing the proportion of cement mortar in the cement-stabilized macadam mixture can enhance the bonding performance of the interface between composite polyurethane composites and cement-stabilized macadam base.

(2) Cohesion work of G1-2, T-1 and TG-1

The cohesion work of nano-modified composite polyurethane composites is numerically equal to twice the surface free energy of nano-modified composite polyurethane composites. The cohesion work of G1-2, T-1 and TG-1 was calculated, and the results are shown in Table 11.

It can be seen from Table 11 that G1-2 has the largest cohesion work.

Consistent with previous studies on polyurethane-based repair materials [36], our results show a negative correlation between cohesion work and material toughness. This trend is mainly attributed to the crosslinking effect of nano-modifiers, which increases the rigidity of the polymer network and reduces its plastic deformation capacity.

**Figure 9:**
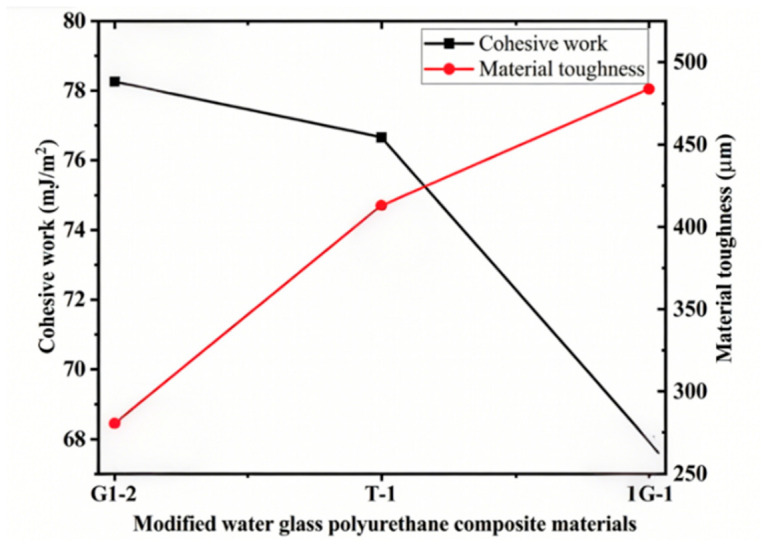
Cohesion work and material toughness of inorganic nano-modified composite polyurethane composites.

It can be seen from Figure 9 that the material toughness of the three nano-modified composite polyurethane composites is negatively correlated with the material cohesion work. The measured data show low variability (coefficients of variation < 5%), confirming the reliability of the results. For better readability, error bars are not displayed in the figure. Material toughness characterizes the tensile properties of the composite within a specific tensile strength threshold (4 MPa), and material cohesion work characterizes the ultimate ability of the composite to resist pull-out failure. Therefore, the data change law in Figure 9 cannot fully prove that there is a direct correlation between the material toughness and cohesion work of the composite. However, it can be indicated that increasing the material toughness of composite polyurethane composites does not necessarily improve the cohesion work of the composites.

(3) Adhesion work of three nano-modified composite polyurethane composites with cement-stabilized macadam mixture

The cement-stabilized macadam mixture base is generally composed of three phases: limestone aggregate, cement mortar and pores, with the proportion of 76.71%:20.57%:2.72%. The adhesion work of nano-modified composite polyurethane composites with cement-stabilized macadam mixture is composed of the adhesion work of nano-modified composite polyurethane composites with limestone aggregate, the adhesion work with cement mortar and the adhesion work with pores, weighted by their composition ratios.

Based on the data in Table 8 and Table 10, the adhesion work of three nano-modified composite polyurethane composites (G1-2, T-1, TG-1) with limestone aggregate, cement mortar and cement-stabilized macadam mixture was calculated, and the calculation results are shown in Table 12.

It can be seen from Table 12 that the adhesion work of the three nano-modified composite polyurethane composites with cement-stabilized macadam mixture is in the order of T-1 > G1-2 > TG-1. Among them, T-1 has a larger adhesion work with cement-stabilized macadam mixture, indicating that carbon nanotube modified composite polyurethane composite has a good effect on enhancing the interface bonding performance between the composite and cement-stabilized macadam mixture. Therefore, from the perspective of enhancing the bonding interface performance between nano-modified composite polyurethane composites and cement-stabilized macadam mixture, it is recommended to use carbon nanotube composite filler modified composite polyurethane composites.

(4) Spalling work of nano-modified composite polyurethane composites with cement-stabilized macadam mixture

The pavement structure base cracks treated by nano-modified composite polyurethane composite grouting will inevitably suffer from water damage. The energy for nano-modified composite polyurethane composites to fall off from the aggregate surface under-water environment is defined as spalling work. According to the formulas and calculations, the spalling work of nano-modified composite polyurethane composites, limestone aggregate, cement mortar and cement-stabilized macadam mixture was obtained, and the results are shown in Table 13.

It can be seen from Table 13 that the spalling work of the three nano-modified composite polyurethane composites with cement-stabilized macadam mixture is in the order of TG-1 > T-1 > G1-2. Among them, the composite polyurethane modified by nano-SiO_2_ and carbon nanotube composite filler (TG-1) has a larger spalling work with limestone aggregate and cement mortar, indicating that the bonding interface between TG-1 and cement-stabilized macadam mixture is more susceptible to water damage, and the bonding interface of nano-SiO_2_ modified composite polyurethane composite is less susceptible to water damage. Statistical analysis confirmed that the differences between the values were significant (*p* < 0.05), supporting the observed ranking.

(5) Energy ratio parameters of nano-modified composite polyurethane composites with cement-stabilized macadam mixture

The energy ratio parameter can evaluate the water stability of nano-modified composite polyurethane composites with cement-stabilized macadam mixture. When its value is greater than 1, it indicates that water has a negative effect on the interface bonding performance between nano-modified composite polyurethane and cement-stabilized macadam, that is, the interface viscosity will decay in the water environment; when the energy ratio parameter is equal to 1, it indicates that water has no effect on the interface bonding performance between nano-modified composite polyurethane and cement-stabilized macadam; when the energy ratio parameter is less than 1, it indicates that water can enhance the interface bonding performance between nano-modified composite polyurethane and cement-stabilized macadam in the water environment.

According to Equation (10), the energy ratio parameters of three nano-modified composite polyurethane composites with cement-stabilized macadam mixture were calculated by substituting the data in Figure 4 and Table 12 and Table 13, as shown in Table 14.

It can be seen from Table 14 that the energy ratio values of the three nano-modified composite polyurethane composites with cement-stabilized macadam mixture are all greater than 1, indicating that water has a negative effect on the interface bonding performance between the three nano-modified composite polyurethanes and cement-stabilized macadam, and water reduces the interface viscosity. The energy ratio of the three nano-modified composite polyurethane composites with cement-stabilized macadam mixture is in the order of G1-2 > T-1 > TG-1, indicating that the composite polyurethane modified by nano-SiO_2_ and carbon nanotube composite filler (TG-1) can better improve the water damage resistance of the composite polyurethane composite.

In summary, the interface bonding performance of the three nano-modified composite polyurethane composites with cement-stabilized macadam mixture is in the order of T-1 > G1-2 > TG-1. Nano-modified composite polyurethane can effectively enhance and improve the interface bonding performance between composite polyurethane composites and cement-stabilized macadam mixture.

### 3.3. Interface Interaction Spectrum of TG-1 and Cement-Stabilized Macadam Mixture

The micro-morphological characteristics of the interface between TG-1 and cement-stabilized macadam base matrix were explained by continuous and systematic observation and analysis of the interface between composite polyurethane and cement-stabilized macadam mixture on the sample by scanning electron microscope (SEM) test [37,38].

**Figure 10:**
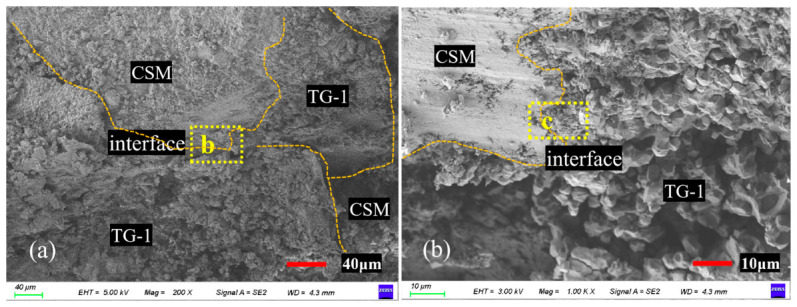

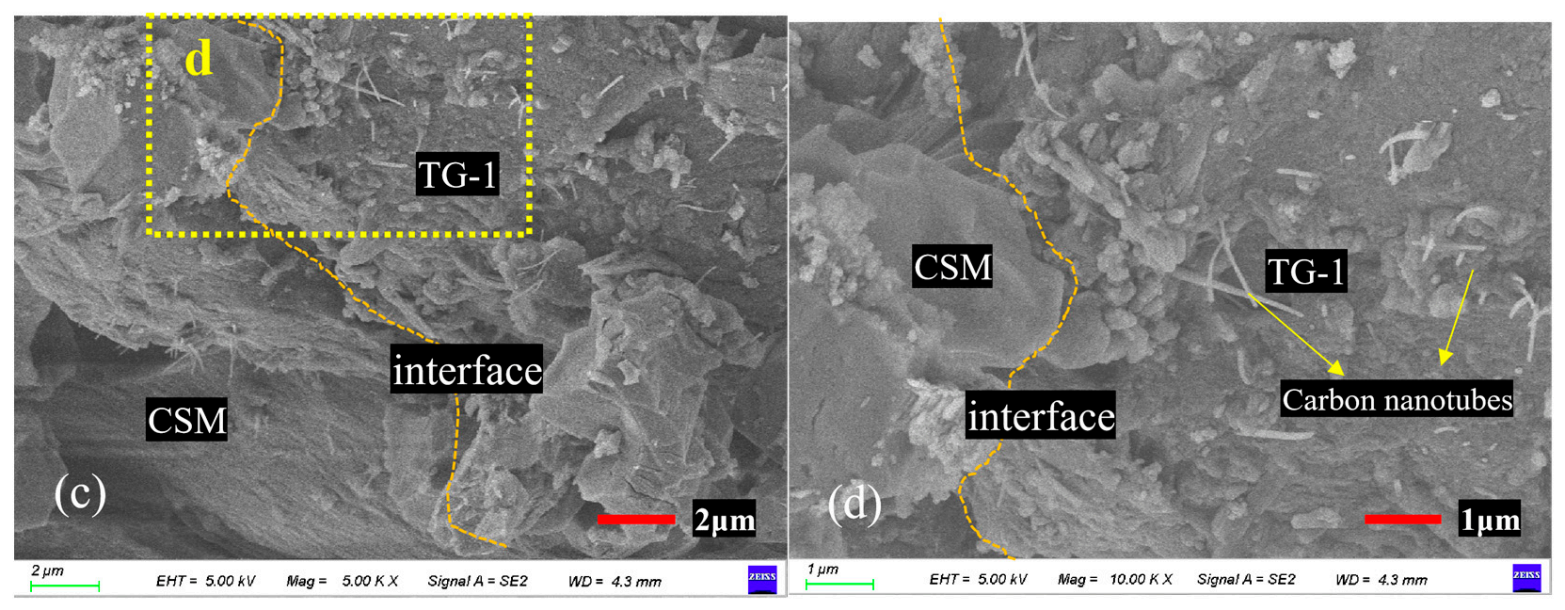
Microscopic images of the interface between TG-1 and cement-stabilized macadam base. (**a**) SEM image at 40 μm scale; (**b**) SEM image at 10 μm scale; (**c**) SEM image at 2 μm scale; (**d**) SEM image at 1 μm scale.

Figure 10 shows the SEM images of the interface between cement-stabilized macadam and TG-1. It can be seen from these images that the cement-stabilized macadam mixture presents a relatively smooth and dense structure, which is the structural characteristic formed by the interaction of cement hydration products and macadam particles, and the TG-1 composite presents a porous and irregular honeycomb structure.

A large number of TG-1 composites at the interface are closely bonded to the surface of cement-stabilized macadam mixture, and TG-1 composites penetrate into the voids of cement-stabilized macadam mixture, wrap the aggregate of cement-stabilized macadam mixture and adhere to its surface. When the magnification is increased, the interface between the two materials can be clearly observed. The aggregate particles are large and the surface is relatively smooth, and the TG-1 material shows different morphologies such as block and needle. The aggregate of cement-stabilized macadam mixture is filled with TG-1 material, forming a good interlocking structure, which can effectively transfer stress and limit the development of cracks.

The observation and analysis of the interface between TG-1 and cement-stabilized macadam mixture are helpful to deeply understand the interaction mechanism of the two materials at the micro scale, and are of great significance for evaluating the interface performance of the two materials, such as bonding strength and durability.

**Figure 11:**
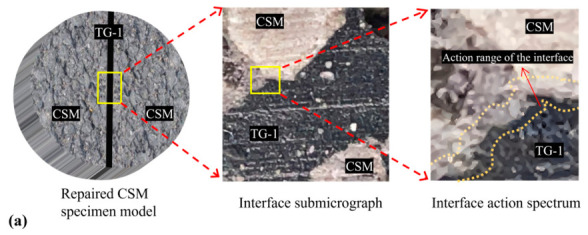

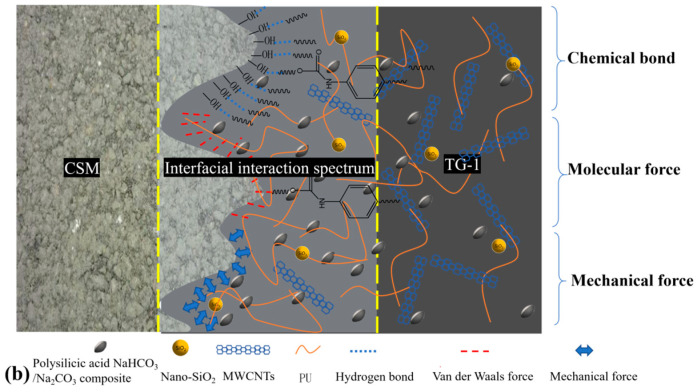
Interaction spectrum of the bonding interface between TG-1 and cement-stabilized macadam mixture ((**a**) CSM repair model and images; (**b**) interface interaction spectrum of TG-1 and CSM).

The interface bonding force between composite polyurethane composite TG-1 and cement-stabilized macadam mixture is not a simple bonding, but a set of energy interaction spectra. The bonding interface mechanism is a complex process, and this interaction spectrum includes several main forces, as shown in Figure 11.

It can be seen from Figure 11 that the interface strength between TG-1 and cement-stabilized macadam mixture mainly comes from chemical bonds, molecular forces, mechanical forces and other aspects. Among them, the polar groups in TG-1 molecules form hydrogen bonds with hydroxyl (-OH) on the surface of cement-stabilized macadam mixture. Hydrogen bonds have low strength but large quantity, which contribute to the bonding strength. In addition, van der Waals forces, including orientation force, induction force and dispersion force, are generated between the molecules at the interface between TG-1 and cement-stabilized macadam mixture. With the increase in the interface contact area, the acting force will be enhanced, which has an impact on the bonding strength. The surface of cement-stabilized macadam mixture usually has a certain roughness, and TG-1 penetrates into the pores and irregularities on the surface of cement-stabilized macadam mixture to form mechanical interlocking, which improves the bonding strength. The bonding interface mechanism between TG-1 and cement-stabilized macadam mixture is the result of the combined action of multiple forces. Chemical bonds, molecular forces and mechanical forces cooperate with each other to jointly determine the strength and durability of the bonding interface. The strength and characteristics of each force will directly affect the bonding performance of the interface between composite polyurethane composites and cement-stabilized macadam mixture. Therefore, strengthening the dominant force can accurately design the interface bonding performance and realize the synergy of the interface interaction spectrum.

## 4. Conclusions

The interface bonding performance between three nano-modified composite polyurethane composites and cement-stabilized macadam mixture was quantitatively evaluated by surface free energy, and the water stability of the interface bonding performance between nano-modified composite polyurethane composites and cement-stabilized macadam mixture was further evaluated by energy ratio parameters.

(1) A preparation method of test specimens for the composition test of cement-stabilized macadam mixture was designed. The cross-section images of cement-stabilized macadam mixture were processed and analyzed by *Image J(v1.54p)* software, and the composition ratios of the three phases (limestone aggregate, cement mortar and pores) of cement-stabilized macadam mixture were analyzed by t-distribution statistics, which provided basic data for the calculation of the interface bonding performance between nano-modified composite polyurethane composites and cement-stabilized macadam mixture.
(2) By calculating the cohesion energy of the three nano-modified composite polyurethane composites, as well as their adhesion work and spalling work with the three components of cement-stabilized macadam mixture, the adhesion work, spalling work and energy ratio of the bonding interface between the three composites and cement-stabilized macadam mixture were calculated by weighting the composition ratio of cement-stabilized macadam mixture, so as to evaluate the interface bonding performance and water stability of nano-modified composite polyurethane composites with cement-stabilized macadam mixture.
(3) The bonding interface mechanism between nano-modified composite polyurethane composites and cement-stabilized macadam mixture is a complex process involving a variety of forces. The interface strength between TG-1 and cement-stabilized macadam mixture is mainly determined by the synergy of chemical bonds, molecular forces and mechanical forces, which jointly determine the strength and durability of the bonding interface.
(4) Through the quantitative evaluation results of the bonding performance of the interface between nano-modified composite polyurethane composites and cement-stabilized macadam mixture, it can be known that the interface bonding performance of the three nano-modified composite polyurethane composites with cement-stabilized macadam mixture is in the order of T-1 > G1-2 > TG-1.

## Figures and Tables

**Table 3:** Aggregate preparation specifications of cement-stabilized macadam mixture.

Specification Name	G2	G8	G11	XG3
Particle size (mm)	20~30	10~20	5~10	0~5

**Table 4:** Aggregate sieving results of four grades of prepared materials.

Specification Name	G2	G8	G11	XG3
Sieve size (mm)	20~30	10~20	5~10	0~5
37.5	100	100	100	100
31.5	100	100	100	100
26.5	82.6	100	100	100
19	17.5	92.4	100	100
16	3.6	58.4	100	100
13.2	1.8	24	100	100
9.5	1	2.3	93.6	100
4.75	0.9	0.7	25.9	100
2.36	0.9	0.6	1.6	91.7
1.18	0.9	0.6	0.8	60.7
0.6	0.9	0.6	0.8	42.9
0.3	0.9	0.6	0.8	22.9
0.15	0.9	0.6	0.8	16.9
0.075	0.9	0.6	0.8	13.3

**Table 5:** Parameter indicators of PO42.5 cement.

Index	Unit	Measured Value	Technical Requirement
Specific surface area	m^2^/kg	371	≥300
Water consumption for normal consistency	%	25.5	≤28.0
Initial setting time	min	231	>180
Final setting time	min	406	360~600
Soundness (Le Chatelier method)	mm	2.4	≤5.0
3 d flexural strength	MPa	5.7	≥4.5
28 d flexural strength	MPa	8.2	≥6.5
3 d compressive strength	MPa	26.0	≥23.0
28 d compressive strength	MPa	48.5	≥42.5

**Table 6:** Surface free energy parameters of two standard liquids.

Name of Standard Liquid	Polar Component (mJ/m^2^)	Dispersion Component (mJ/m^2^)	Surface Free Energy (mJ/m^2^)
Distilled water	43.7	29.1	72.8
Diiodomethane	1.3	49.5	50.8

**Table 7:** Contact angles between nano-modified composite polyurethane composites and standard liquids.

Name of Composite Polyurethane Composite	Distilled Water		Diiodomethane	
	Average Value (°)	Variation Coefficient (%)	Average Value (°)	Variation Coefficient (%)
G1-2	77.92	0.97	42.12	3.04
T-1	76.07	1.01	44.85	4.39
TG-1	77.97	2.23	53.95	3.26

**Table 8:** Surface free energy parameters of three nano-modified composite polyurethane composites.

Name of Composite Polyurethane Composite	Polar Component (mJ/m^2^)	Dispersion Component (mJ/m^2^)	Surface Free Energy (mJ/m^2^)
G1-2	3.10 ± 0.27	36.04 ± 1.15	39.13 ± 1.21
T-1	4.29 ± 0.44	34.04 ± 1.54	38.33 ± 1.66
TG-1	5.18 ± 0.50	28.79 ± 1.13	33.97 ± 1.20

**Table 9:** Contact angles between limestone aggregate, cement mortar and standard liquids.

Name of Material	Distilled Water		Diiodomethane	
	Average Value (°)	Variation Coefficient (%)	Average Value (°)	Variation Coefficient (%)
Limestone	74.24	1.21	60.80	7.48
Cement mortar	47.60	2.75	35.88	5.31

**Table 10:** Surface free energy parameters of limestone aggregate and cement mortar.

Name of Material	Polar Component (mJ/m^2^)	Dispersion Component (mJ/m^2^)	Surface Free Energy (mJ/m^2^)
Limestone aggregate	9.11 ± 0.21	23.84 ± 0.28	32.95 ± 0.35
Cement mortar	20.06 ± 0.30	33.75 ± 0.24	53.81 ± 0.38

**Table 11:** Cohesion work of three composite polyurethane composites.

Name of Composite Polyurethane Composite	Cohesion Work (mJ/m^2^)
G1-2	78.26
T-1	76.66
TG-1	67.94

**Table 12:** Adhesion work of three nano-modified composite polyurethane composites with cement-stabilized macadam mixture and its components.

Name of Composite Polyurethane Composite	Limestone (mJ/m^2^)	Cement Mortar (mJ/m^2^)	Cement-Stabilized Macadam Mixture (mJ/m^2^)
G1-2	69.25 ± 0.24	85.52 ± 0.28	72.84 ± 0.21
T-1	69.48 ± 0.27	86.34 ± 0.31	73.14 ± 0.24
TG-1	66.14 ± 0.30	82.73 ± 0.34	69.60 ± 0.27

**Table 13:** Spalling work of three nano-modified composite polyurethane composites with cement-stabilized macadam mixture and its components.

Name of Composite Polyurethane Composite	Grouting Material (mJ/m^2^)	Limestone (mJ/m^2^)	Cement Mortar (mJ/m^2^)	Cement-Stabilized Macadam Mixture (mJ/m^2^)
G1-2	88.05	33.12 ± 0.30	46.16 ± 0.35	32.50 ± 0.27
T-1	90.33	36.78 ± 0.33	49.22 ± 0.38	38.34 ± 0.30
TG-1	87.98	46.49 ± 0.36	59.20 ± 0.41	47.84 ± 0.33

**Table 14:** Energy ratio of three nano-modified composite polyurethane composites with cement-stabilized macadam mixture.

Name of Composite Polyurethane Composite	Cement-Stabilized Macadam Mixture
G1-2	2.24
T-1	1.91
TG-1	1.45

## Data Availability

The raw experimental data supporting the findings of this study are available from the corresponding author upon reasonable request.
